# Clinical features and gut microbiota alterations in hypertensive individuals with left ventricular hypertrophy

**DOI:** 10.3389/fmicb.2025.1647668

**Published:** 2025-11-12

**Authors:** Liang Cai, Guo-Qiang Liu, Chun-Mei Zeng, Wen-Feng Xu, Ri-Na Jiang, Hai-Yan Chen

**Affiliations:** 1 Guangxi Medical University, Nanning, Guangxi, China; 2 Department of Cardiology, Yulin First People’s Hospital, The Sixth Affiliated Hospital of Guangxi Medical University Yulin, Yulin, Guangxi, China; 3 Center for Genomic and Personalized Medicine, Guangxi Key Laboratory for Genomic and Personalized Medicine, Guangxi Collaborative Innovation Center for Genomic and Personalized Medicine, Guangxi Medical University, Nanning, Guangxi, China; 4 Department of Ultrasound, Yulin First People’s Hospital, The Sixth Affiliated Hospital of Guangxi Medical University Yulin, Yulin, Guangxi, China

**Keywords:** clinical indicators, high-throughput sequencing, hypertension, gut microbiota, left ventricular hypertrophy

## Abstract

**Objective:**

This study aims to identify potential diagnostic biomarkers for individuals with hypertension and left ventricular hypertrophy (LVH) by characterizing associated clinical features and gut microbiota profiles.

**Methods:**

Participants were classified into three groups: a hypertrophy group (hypertension with LVH, *n* = 63), a non-hypertrophy group (hypertension without LVH, *n* = 64), and a control group (healthy participants, *n* = 33). Clinical parameters were recorded, and fecal samples were analyzed for microbial diversity, abundance, and distribution.

**Results:**

Patients in the hypertrophy group exhibited elevated body mass index (BMI), uric acid, triglycerides, and homocysteine levels, as well as reduced estimated glomerular filtration rate and high-density lipoprotein cholesterol. Structural cardiac changes were more pronounced in this group. Increased BMI and γ-glutamyl transferase levels emerged as independent risk factors for LVH. A significant reduction in Actinobacteria abundance was observed in the hypertrophy group compared to healthy controls. At the genus level, microbial compositions in the non-hypertrophy and control groups were more similar. *Blautia* was significantly enriched in patients with LVH, while *Streptococcus equinus* showed increased abundance at the species level (*p* < 0.05). The area under the receiver operating characteristic curve for gut microbiota markers in distinguishing patients with LVH from healthy controls was 0.839, and 0.796 when differentiating from patients with hypertension but without LVH.

**Conclusion:**

Patients with hypertension and LVH demonstrated distinct metabolic abnormalities and alterations in gut microbiota composition. These findings reported potential microbial biomarkers and pathways for the prevention and management of hypertension-related target organ damage.

## 1 Introduction

Long-term uncontrolled hypertension is a major contributor to organ damage, particularly affecting the heart, brain, and kidneys. Among these, left ventricular hypertrophy (LVH) represents one of the most prevalent complications and serves as an independent risk factor for cardiovascular events. According to the World Health Organization Global Hypertension Report 2023, the global prevalence of hypertension rose from 650 million to 1.3 billion between 1990 and 2019, with over 30% of individuals with hypertension exhibiting LVH. The severity of LVH is strongly associated with increased cardiovascular disease incidence and elevated mortality risk (Leache et al., 2021).

Recent studies have shown that gut microbiota dysbiosis is not only closely associated with the development of hypertension, but may also contribute to the onset and progression of LVH through multiple mechanisms (Liu et al., 2018; Qu et al., 2022; Cao et al., 2024). These mechanisms involve hemodynamic changes, neuroendocrine activation, inflammatory cell infiltration and cytokine release, oxidative stress, and genetic predisposition (Djekic et al., 2020). However, population-based data concerning the clinical and gut microbiota characteristics of individuals with both hypertension and LVH remain limited.

Comprehensive assessment of clinical profiles and gut microbiota distribution in this population is therefore critical for early identification and prevention of LVH progression, with the potential to improve cardiovascular outcomes. This study examined the clinical indicators and gut microbiota profiles of patients with hypertension and LVH, and assessed their association with the severity of LVH. The findings aim to provide population-level evidence supporting the mechanistic understanding of hypertension-induced cardiac target organ damage, and to inform preventive and early therapeutic strategies for affected individuals.

## 2 Materials and methods

### 2.1 General data

A total of 127 patients diagnosed with hypertension at the First People’s Hospital of Yulin City between April 2024 and December 2024 were consecutively enrolled. As determined by cardiac ultrasound findings, participants were categorized into a hypertrophy group (hypertension with left ventricular hypertrophy, *n* = 63) and a non-hypertrophy group (hypertension without left ventricular hypertrophy, *n* = 64). Additionally, healthy individuals undergoing routine physical examinations during the same period were recruited as the control group (*n* = 33).

General demographic data, medical history, lifestyle behaviors, laboratory test results, auxiliary examination findings, and medication use were documented for all participants. Stool samples were collected, and the composition, diversity, and abundance of gut microbiota were analyzed using high-throughput sequencing of amplicons targeting the V3–V4 region of the 16S rRNA gene.

Ethical approval was obtained from the Ethics Committee of the First People’s Hospital of Yulin City (YLSY-IRB-RP-2023022), and written informed consent was obtained from all participants.

### 2.2 Diagnostic criteria for left ventricular hypertrophy

The left ventricular mass index (LVMI) serves as a primary echocardiographic indicator for the diagnosis of LVH, defined by an LVMI of ≥115 g/m^2^ in males and ≥95 g/m^2^ in females (Mancia et al., 2013). End-diastolic left ventricular internal diameter (LVID), interventricular septal thickness (IVST), and left ventricular posterior wall thickness (LVPWT) are measured, and left ventricular mass (LVM) is calculated accordingly. The LVM is then normalized to body surface area (BSA) to obtain the LVMI. The calculation formula is as follows:


$$\mathrm{LVM}\;(\mathrm{mg})=0.8\times 1.04\times [{\left(\text{LVID}+\text{IVST}+\text{LVPWT}\right)}^{3}-{\text{LVID}}^{3}]+0.6$$

$$\text{LVMI}\;(g/m^{2})=\mathrm{LVM}/\mathrm{BSA}$$

$$\begin{array}{l} \text{Body surface area}\;\mathrm{BSA}\;(m^{2})=0.007184\times \text{height}\;{\left(\mathrm{cm}\right)}^{0.725}\\ \times \text{weight}\;{\left(\mathrm{kg}\right)}^{0.425} \end{array}$$

### 2.3 Inclusion and exclusion criteria

#### 2.3.1 Inclusion criteria

Age ≥18 years; diagnosis of primary hypertension based on established clinical guidelines (Hypertension is diagnosed if clinic blood pressure is ≥ 140/90 mmHg on three separate days without antihypertensive medication or home blood pressure monitoring shows an average blood pressure ≥135/85 mmHg over 5 to 7 consecutive days. Patients with a history of hypertension currently taking antihypertensive medication should still be diagnosed with hypertension, even if their blood pressure is below the above diagnostic thresholds); provision of written informed consent.

#### 2.3.2 Exclusion criteria

Absence of a stool sample; diagnosis of secondary hypertension; use of antibiotics or probiotics within 4 weeks prior to stool collection; history of chronic diarrhea lasting more than 1 month, enteritis, or other gastrointestinal diseases; history of radiotherapy or chemotherapy for intestinal or other malignancies; history of long-term heavy alcohol consumption; pregnancy.

### 2.4 Stool collection

Fresh stool specimens were self-collected by participants using sterile collection tubes pre-chilled at 4 °C. Participants were instructed to collect mid-portion stool (avoiding urine/water contact) immediately after natural defecation, and transfer approximately 100 mg feces into the tube using the provided sterile spatula. The tube was tightly capped and vigorously shaken for 15 s to homogenize the sample with DNA stabilization buffer. All samples were labeled with unique IDs and time of collection, then stored in portable 4 °C coolers with ice packs. Within 2 h, samples were transported to the laboratory, logged into the sample management system, and temporarily stored at −40 °C in a monitored freezer (single-layer placement, ≤24 h). For long-term preservation, samples were transferred on dry ice to designated positions within −80 °C ultra-low freezers.

### 2.5 16S rDNA sequencing and bioinformatics analysis

The stool samples were transported on dry ice to the testing institution (Wuhan Metware Biotechnology Inc.) for 16S rDNA sequencing and subsequent bioinformatics analysis.

Genomic DNA was extracted from samples using either the CTAB or SDS method. The purity and concentration of the extracted DNA were assessed via agarose gel electrophoresis. Qualified DNA was diluted to 1 ng/μL with sterile water. This diluted DNA served as the template for PCR amplification targeting specific hypervariable regions. Amplification was performed using barcoded primers, Phusion^®^ High-Fidelity PCR Master Mix with GC Buffer (New England Biolabs), and a high-efficiency, high-fidelity polymerase to ensure amplification efficiency and accuracy. PCR products were verified by electrophoresis on a 2% agarose gel. Qualified amplicons were purified using magnetic beads, quantified using a microplate spectrophotometer, and pooled in equimolar amounts based on concentration. The pooled product was re-checked on a 2% agarose gel, and the target bands were excised and purified using a gel extraction kit (Qiagen). Sequencing libraries were constructed using the TruSeq^®^ DNA PCR-Free Sample Preparation Kit. The quality and concentration of the final libraries were assessed using Qubit fluorometry and qPCR. Qualified libraries were sequenced on the Illumina NovaSeq 6000 platform.

Raw sequencing reads were demultiplexed based on their unique barcode and primer sequences, followed by the removal of these adapter sequences. Sequence quality control was performed using fastp^1^ to obtain high-quality reads. Paired-end reads were then assembled into longer, high-quality fragments (clean tags) using FLASH.^2^ Potential chimeric sequences were identified and removed from the clean tags by comparing them against a reference database using vsearch,^3^ resulting in effective tags. All effective tags across samples were clustered into operational taxonomic units (OTUs) at 97% sequence identity using the UPARSE algorithm within USEARCH.^4^ Alternatively, amplicon sequence variants (ASVs) were generated using a denoising method. Taxonomic annotation of OTUs/ASVs was performed against the SILVA SSU rRNA database^5^ using the Mothur method with a confidence threshold of 0.8 to 1.0. Community composition was subsequently analyzed at each taxonomic level.

### 2.6 Statistical analysis

Statistical analysis was conducted using SPSS version 27.0. Categorical variables were presented as counts and percentages (*n*, %), and comparisons between groups were performed using either the chi-square test or Fisher’s exact test, as appropriate. Continuous variables with normal distribution were expressed as mean ± standard deviation (SD). Comparisons among three groups were performed using one-way analysis of variance, while comparisons between two groups were conducted using the independent samples *t*-test. For continuous variables not conforming to a normal distribution, non-parametric tests were applied for group comparisons.

Multivariate logistic regression analysis was used to assess the association between relevant clinical indicators and the presence of LVH. The phyloseq and vegan packages in R were used to calculate alpha diversity indices, including Shannon, Simpson, ACE, and Chao1. Dilution and species accumulation curves were generated using R software. Differences in alpha diversity indices among groups were analyzed, with the Kruskal–Wallis test applied for comparisons across the three groups.

For beta diversity analysis, the Jaccard distance was computed using the phyloseq package, and principal coordinate analysis (PCoA) plots were generated. Differences in beta diversity among groups were assessed using the Kruskal–Wallis test. Linear discriminant analysis effect size (LEfSe) was used to compare the relative abundance of bacteria at the genus level between groups, with the threshold for LDA score set at 4. Differential bacterial taxa between groups were identified through LEfSe analysis.

A random forest prediction model was constructed to assess the discriminatory capacity of microbial features, and receiver operating characteristic curves were plotted accordingly. All statistical tests were two-sided, and a value of *p* < 0.05 was considered statistically significant.

## 3 Results

### 3.1 Clinical characteristics of the study participants

A total of 160 stool samples were consecutively collected from 63 patients with hypertension and left ventricular hypertrophy (hypertrophy group, *n* = 63), 64 patients with hypertension without LVH (non-hypertrophy group, *n* = 64), and 33 healthy individuals (control group, *n* = 33).

Detailed clinical characteristics of the three groups are presented in Table 1. The mean age of participants was 51.9 ± 14.9 years, with 85 participants (53.1%) being male. The proportion of male participants was higher in the hypertrophy group. Body mass index (BMI) (*p* < 0.001; *p* = 0.001), uric acid (*p* < 0.001; *p* = 0.013), triglycerides (*p* = 0.015; *p* = 0.028), homocysteine (*p* = 0.025; *p* = 0.035), alkaline phosphatase (*p* = 0.013; *p* = 0.037), and γ-glutamyl transferase (*p* = 0.005; *p* < 0.001) levels were significantly elevated in the hypertrophy group compared to the control and non-hypertrophy groups. In contrast, estimated glomerular filtration rate (*p* < 0.001; *p* < 0.001) and high-density lipoprotein cholesterol (HDL-C) (*p* = 0.003; *p* = 0.038) levels were significantly lower. With respect to cardiac structural parameters, the left atrial anteroposterior diameter (*p* < 0.001; *p* < 0.001), right ventricular internal diameter (*p* = 0.019; *p* = 0.026), and E/e′ ratio (*p* < 0.001; *p* = 0.005) were significantly increased in the hypertrophy group when compared to the control and non-hypertrophy groups. Additionally, the use of calcium channel blockers (57.1%, *p* = 0.027) and statins (73.0%, *p* = 0.013) was more frequent in the hypertrophy group.

**Table 1:** Characteristics of clinical data.

Variables	Control group (*n* = 33)	Non-hypertrophy group (*n* = 64)	Hypertrophy group (*n* = 63)	P1	P2	P3	P
Sex [male (%)]	12 (36.4)	26 (40.6)	47 (74.6)	0.684	<0.001	<0.001	<0.001
Age (year)	49.4 ± 12.2	50.1 ± 15.4	55.0 ± 15.3	0.833	0.072	0.072	0.098
BMI (kg/m^2^)	22.0 ± 3.8	24.1 ± 3.5	26.5 ± 4.3	0.009	<0.001	0.001	<0.001
Waist circumference (WC) (cm)	82.5 ± 8.5	88.2 ± 10.3	92.3 ± 8.9	0.125	<0.001	0.235	<0.001
Smoking [*n* (%)]	6 (18.2)	10 (15.6)	17 (27)	0.748	0.337	0.118	0.265
Drinking [*n* (%)]	4 (12.1)	7 (10.9)	12 (19)	0.862	0.387	0.200	0.393
Family history [*n* (%)]	2 (6.1)	14 (21.9)	14 (31.7)	0.047	0.004	0.209	0.016
Diabetes [*n* (%)]	—	7 (11.3)	8 (12.7)	—	—	0.809	—
Cerebrovascular diseasen [*n* (%)]	—	4 (6.5)	3 (4.8)	—	—	0.681	—
Renal insufficiency [*n* (%)]	—	3 (5.0)	13 (21.0)	—	—	0.009	—
SBP (mmHg)	121.1 ± 14.0	151.5 ± 22.2	157.9 ± 26.0	<0.001	<0.001	0.142	<0.001
DBP (mmHg)	79.7 ± 8.0	93.9 ± 17.9	98.8 ± 20.1	<0.001	<0.001	0.152	<0.001
Heart rate (bpm)	79.3 ± 12.5	80.2 ± 13.2	82.4 ± 15.9	0.768	0.332	0.380	0.518
Fasting blood glucose (FBG) (mmol/L)	5.1 ± 0.8	5.3 ± 1.2	5.7 ± 2.4	0.344	0.058	0.209	0.173
Uric acid (umol/L)	346.4 ± 74.1	374.4 ± 119.5	425.3 ± 108.8	0.158	<0.001	0.013	0.001
Urea nitrogen (mmol/L)	4.8 ± 1.3	4.7 ± 1.7	5.8 ± 3.0	0.807	0.075	0.014	0.018
Creatinine (umol/L)	68.9 ± 18.1	80.0 ± 21.2	131.4 ± 200.3	0.012	0.077	0.047	0.027
eGFR (ml/min/1.73m²)	113.0 ± 46.7	93.2 ± 25.8	77.4 ± 26.7	0.008	<0.001	<0.001	<0.001
TC (mmol/L)	4.6 ± 1.0	4.8 ± 1.0	4.9 ± 1.1	0.388	0.139	0.398	0.303
TG (mmol/L)	1.5 ± 1.1	1.7 ± 1.1	2.3 ± 1.7	0.296	0.015	0.028	0.011
HDL (mmol/L)	1.2 ± 0.3	1.2 ± 0.3	1.0 ± 0.3	0.350	0.003	0.038	0.014
LDL (mmol/L)	2.8 ± 0.9	2.9 ± 0.8	3.0 ± 0.9	0.493	0.323	0.687	0.599
Homocysteine (umol/L)	10.1 ± 4.0	11.2 ± 3.2	13.3 ± 7.1	0.175	0.025	0.035	0.013
Alanine (U/L)	11.8 ± 6.5	16.8 ± 12.0	20.4 ± 21.2	0.009	0.004	0.239	0.039
Asparaginic acid (U/L)	23.6 ± 5.0	21.0 ± 5.5	23.7 ± 8.5	0.024	0.951	0.034	0.052
Alkaline phosphatase (U/L)	69.4 ± 23.9	72.8 ± 24.8	81.4 ± 21.1	0.512	0.013	0.037	0.028
γ-glutamyl transferase (U/L)	28.8 ± 17.6	30.7 ± 19.0	48.8 ± 37.9	0.640	0.005	<0.001	<0.001
Total bile acids (umol/L)	7.3 ± 5.0	5.2 ± 3.0	8.1 ± 9.3	0.030	0.671	0.024	0.046
Hemoglobin (g/L)	129.0 ± 18.7	132.1 ± 27.4	140.6 ± 20.2	0.546	0.007	0.050	0.034
Glycosylated hemoglobin (%)	5.9 ± 0.5	6.0 ± 0.8	6.3 ± 1.1	0.973	0.183	0.042	0.075
Urinary casts [*n* (%)]	3 (9.1)	10 (15.6)	6 (9.5)	0.357	0.945	0.300	0.487
Urine protein quantification (mg/L)	100.7 ± 26.0	132.3 ± 128.2	195.1 ± 264.1	0.521	0.352	0.122	0.206
Urine microalbumin (mg/L)	6.4 ± 4.4	22.1 ± 49.0	66.0 ± 156.6	0.404	0.321	0.044	0.085
Urine total protein-creatinine ratio (mg/g)	80.0 ± 41.5	113.1 ± 68.3	251.8 ± 501.8	0.217	0.372	0.042	0.097
Urine microalbumin-creatinine ratio (mg/g)	4.9 ± 4.0	16.8 ± 27.3	95.3 ± 283.3	0.258	0.405	0.037	0.085
Cardiac ultrasound							
Inner diameter of the aortic root (mm)	20.2 ± 1.7	21.6 ± 7.6	21.5 ± 1.7	0.320	<0.001	0.943	0.414
Left atrial anteroposterior diameter (mm)	29.7 ± 3.0	31.1 ± 5.1	34.2 ± 4.4	0.135	<0.001	<0.001	<0.001
Left atrial horizontal diameter (mm)	29.7 ± 3.0	39.7 ± 12.7	36.9 ± 8.6	0.015	<0.001	0.454	<0.001
Right atrial anteroposterior diameter (mm)	41.7 ± 4.2	41.4 ± 4.1	43.5 ± 5.1	0.728	0.077	0.010	0.022
Right atrial horizontal diameter (mm)	34.3 ± 4.2	33.9 ± 5.0	34.8 ± 4.7	0.686	0.622	0.303	0.571
Right ventricular internal diameter (mm)	19.9 ± 1.9	20.2 ± 1.7	21.0 ± 2.2	0.420	0.019	0.026	0.017
End-diastolic left ventricular internal diameter (mm)	42.8 ± 3.6	43.9 ± 4.9	45.4 ± 3.8	0.235	0.001	0.054	0.011
Interventricular septal thickness (mm)	9.1 ± 1.1	9.5 ± 0.9	12.7 ± 1.2	0.119	<0.001	<0.001	<0.001
Left ventricular posterior wall thickness (mm)	9.0 ± 0.8	9.4 ± 0.9	12.0 ± 1.5	0.040	<0.001	<0.001	<0.001
LVMI (g/m²)	78.8 ± 17.1	82.8 ± 13.9	117.1 ± 30.5	0.221	<0.001	<0.001	<0.001
EF (%)	67.1 ± 4.9	66.9 ± 9.1	65.0 ± 7.9	0.881	0.166	0.217	0.315
FS (%)	37.5 ± 4.1	37.6 ± 6.4	36.1 ± 5.3	0.941	0.195	0.169	0.290
EDV (ml)	83.5 ± 16.2	88.6 ± 24.4	95.0 ± 19.5	0.287	0.005	0.105	0.034
ESV (ml)	27.7 ± 7.4	31.6 ± 18.5	35.8 ± 20.0	0.319	0.040	0.392	0.141
SV (ml)	55.6 ± 12.0	56.1 ± 12.3	60.5 ± 11.3	0.864	0.094	0.153	0.185
E/e′	8.9 ± 1.8	10.1 ± 3.1	11.8 ± 3.4	0.018	<0.001	0.005	<0.001
E/A [*n* (%)]				0.213	0.021	0.197	0.069
>1	16 (51.6)	24 (38.1)	16 (27.1)				
<1	15 (48.4)	39 (61.9)	43 (72.9)				
e′/a′ [*n* (%)]				0.105	0.004	0.142	0.017
>1	10 (32.3)	11 (17.5)	5 (8.5)				
<1	21 (67.7)	52 (82.5)	54 (91.5)				
ECG ST-T changes [*n* (%)]	5 (15.6)	19 (31.7)	28 (45.2)	0.095	0.004	0.126	0.015
Carotid plaque [*n* (%)]				0.034	0.002	0.056	0.004
Single	11 (42.3)	13 (21.3)	16 (25.8)				
Multiple	3 (11.5)	22 (36.1)	32 (51.6)				
Maximum plaque area (mm²)	19.3 ± 17.8	32.7 ± 23.6	41.1 ± 44.5	0.062	0.080	0.312	0.117
Maximum plaque length (mm)	8.4 ± 4.2	11.9 ± 5.1	15.0 ± 11.3	0.031	0.039	0.102	0.036
Maximum plaque width (mm)	2.0 ± 0.7	2.6 ± 0.8	2.5 ± 1.2	0.037	0.133	0.907	0.205
Medications [*n* (%)]							
CCB	—	24 (37.5)	36 (57.1)	—	—	0.027	—
ACEI/ARB/ARNi	—	43 (67.2)	46 (73.0)	—	—	0.473	—
Diuretics	—	11 (17.5)	6 (9.5)	—	—	0.192	—
β receptor blockers	—	16 (25.0)	24 (38.1)	—	—	0.112	—
Statins	—	33 (51.6)	46 (73.0)	—	—	0.013	—

P1: non-hypertrophy group vs. control group; P2: hypertrophy group vs. control group; P3: non-hypertrophy group vs. hypertrophy group; P: comparison between three groups.

### 3.2 Multivariate analysis of LVH

LVH was set as the dependent variable (2 = hypertension with LVH, 1 = hypertension without LVH), and variables including sex, BMI, renal function, blood lipid levels, uric acid, and homocysteine were included as independent variables in a multivariate logistic regression analysis. An increase in BMI and γ-glutamyl transferase levels were identified as independent risk factors for LVH [BMI: OR = 1.316, 95% CI: 1.089–1.590, *p* = 0.004; γ-glutamyl transferase: OR = 1.026, 95% CI: 1.003–1.051, *p* = 0.029] (Table 2).

**Table 2:** Results of multivariate logistic regression analysis of LVH.

Variables	*B*	SE	Wald	*p*-value	OR value	95% CI	
						Lower limit	Upper limit
Sex	−1.408	0.723	3.792	0.052	0.245	0.059	1.009
BMI	0.275	0.097	8.081	0.004	1.316	1.089	1.590
Uric acid	−0.004	0.004	1.481	0.224	0.996	0.988	1.003
Urea nitrogen	0.057	0.237	0.057	0.811	1.058	0.665	1.683
Blood creatinine	−0.003	0.017	0.026	0.871	0.997	0.964	1.031
eGFR	−0.039	0.020	3.657	0.056	0.962	0.924	1.001
Triglycerides	−0.170	0.265	0.410	0.522	0.844	0.502	1.419
High-density lipoprotein cholesterol	−1.085	1.118	0.942	0.332	0.338	0.038	3.023
Homocysteine	0.039	0.065	0.365	0.546	1.040	0.915	1.182
Asparaginic acid	0.059	0.056	1.108	0.292	1.061	0.950	1.185
Alkaline phosphatase	0.007	0.014	0.287	0.592	1.007	0.981	1.034
γ-glutamyl transferase	0.026	0.012	4.781	0.029	1.026	1.003	1.051
Total bile acids	0.118	0.083	2.029	0.154	1.125	0.957	1.324
Glycosylated hemoglobin	0.471	0.350	1.816	0.178	1.602	0.807	3.178
Urine total protein-creatinine ratio	0.007	0.006	1.124	0.289	1.007	0.994	1.020
Urine microalbumin-creatinine ratio	−0.001	0.014	0.005	0.946	0.999	0.972	1.027

### 3.3 Gut microbiota analysis

#### 3.3.1 Results of operational taxonomic units (OTUs) analysis of gut microbiota

**Figure 1:**
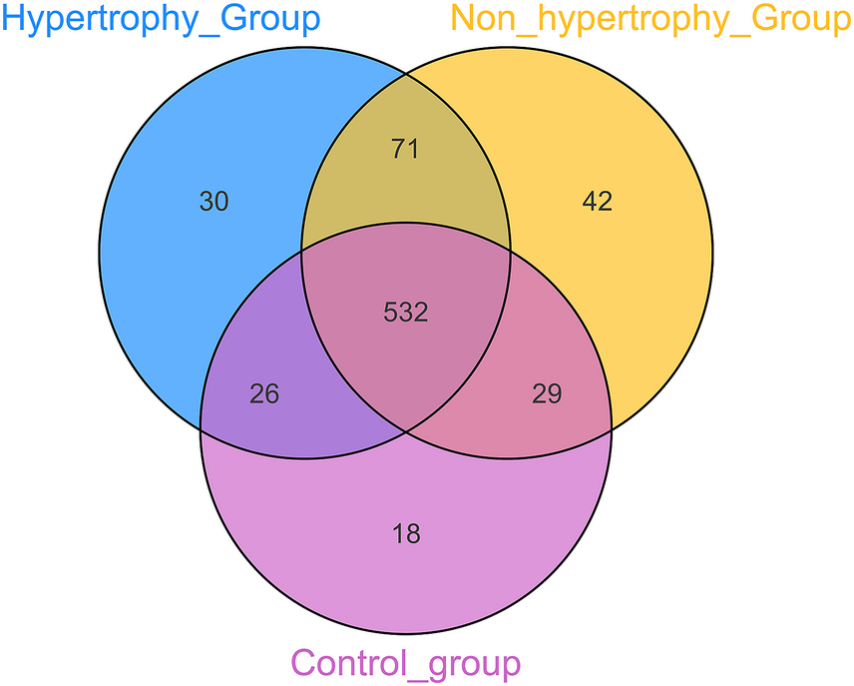
Venn diagrams of the OTU of intestinal flora among different groups.

OTUs were clustered at a 97% sequence similarity threshold, and the intersection of OTUs among the three groups was analyzed. A Venn diagram depicted that a total of 532 OTUs were identified across all groups, with 18 unique to the control group, 42 unique to the non-hypertrophy group, and 30 unique to the hypertrophy group (Figure 1). The number of sequencing reads generated for each sample were provided in Supplementary Table S1. The sequencing data were deposited to Metware Cloud with project number MWY-24-7280.^6^ All data generated in this study are available on reasonable request.

#### 3.3.2 Alpha diversity analysis

**Figure 2:**
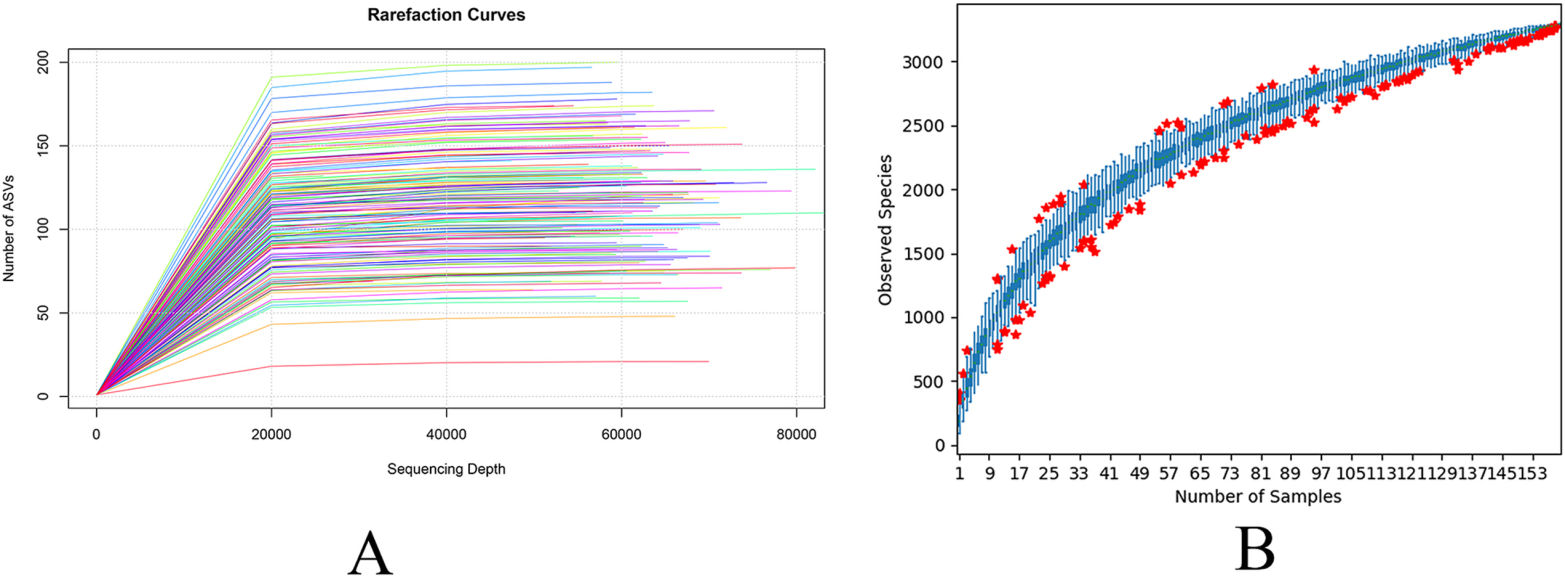
Dilution curves **(A)** and species accumulation box plots **(B)** of sequencing data.

To assess the adequacy of the sample size for capturing species richness, dilution curves and species accumulation box plots were generated by progressively increasing the sequencing depth through random sampling (Figures 2A,B). The observed plateau in the curves with increasing sample size showed that the sequencing depth was sufficient, and the data were stable.

**Figure 3:**
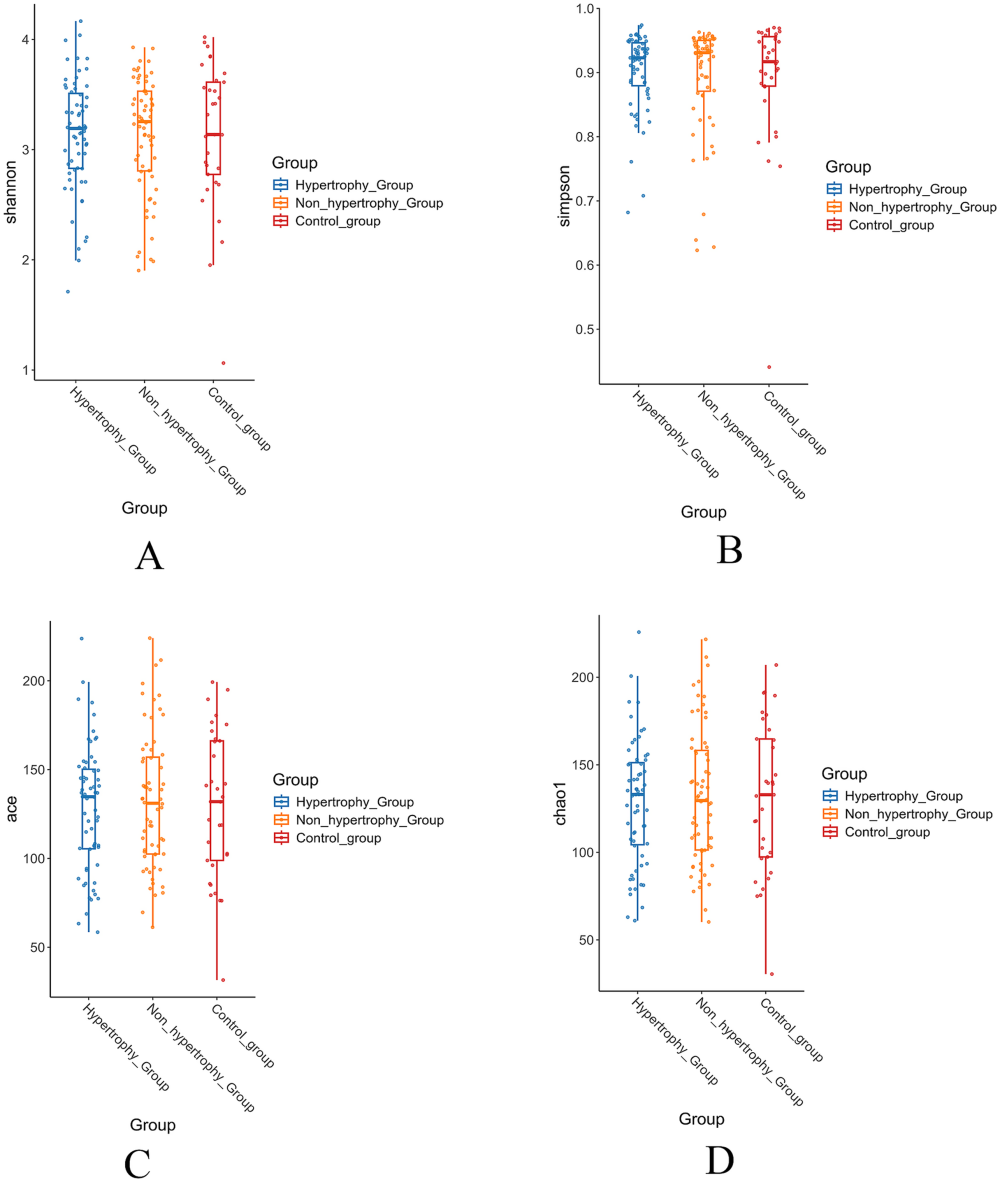
The results of alpha diversity of intestinal flora in each group (**A**: Shannon index; **B**: Simpson index; **C**: Ace index; **D**: Chao1 index).

Alpha diversity of the gut microbiota was assessed among the hypertrophy, non-hypertrophy, and control groups using the Shannon, Simpson, ACE, and Chao1 indices. No statistically significant differences in alpha diversity were observed among the three groups (*p* > 0.05) (Figures 3A–D).

#### 3.3.3 Beta diversity analysis

**Figure 4:**
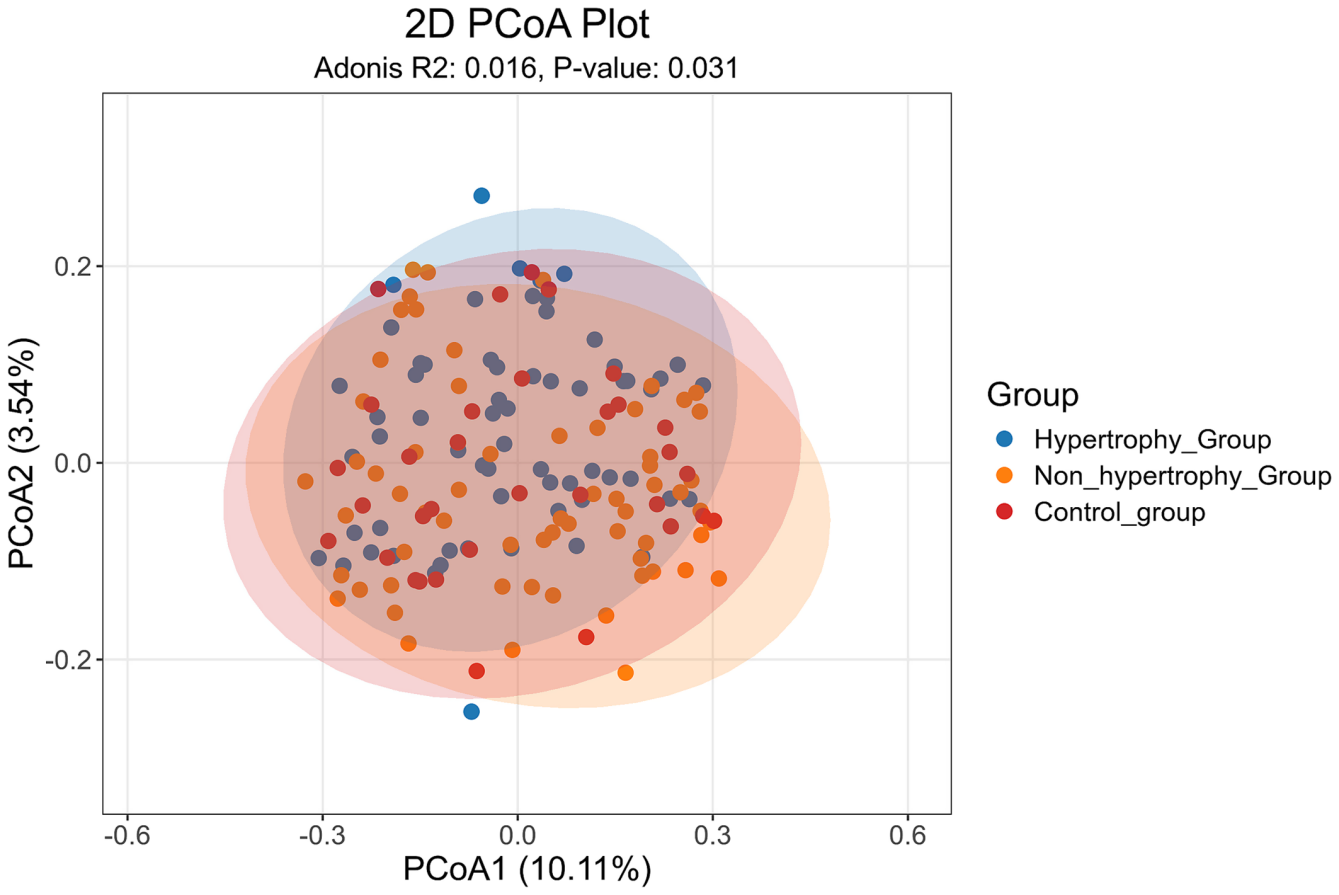
Score charts of PCoA analysis of intestinal flora.

PCoA was conducted on all samples using the Jaccard distance algorithm to assess beta diversity differences among the hypertrophy, non-hypertrophy, and control groups. The results (Figure 4) showed a statistically significant difference in β diversity among the three groups (Adonis: *R*^2^ = 0.016, *p* = 0.031).

**Figure 5:**
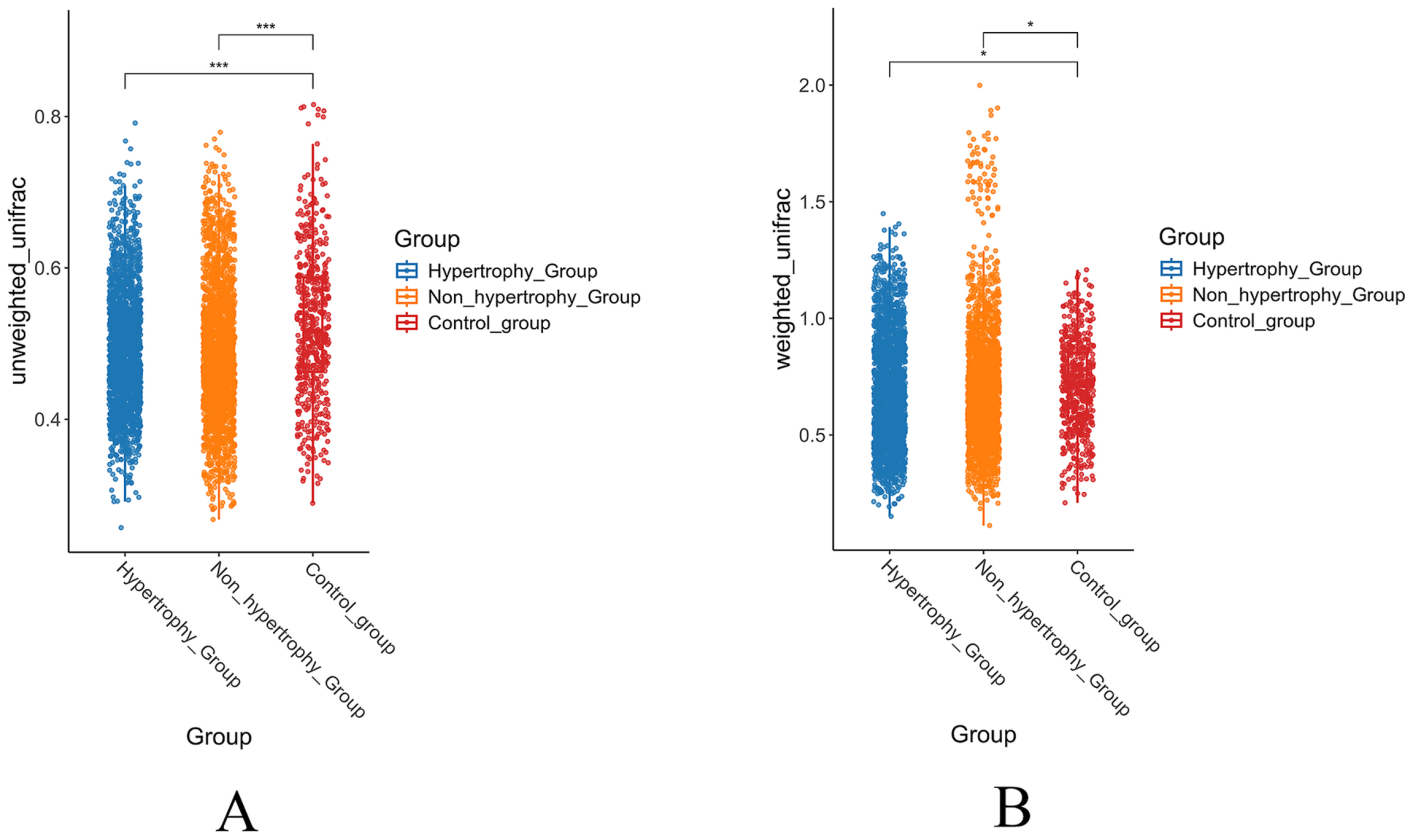
The results of beta diversity of intestinal flora in each group (**A**: unweighted UniFrac distance; **B**: weighted UniFrac distance).

To further assess intergroup differences in beta diversity, both unweighted and weighted UniFrac distance algorithms were applied. The unweighted UniFrac analysis showed statistically significant differences in beta diversity between the hypertrophy and control groups, as well as between the non-hypertrophy and control groups (*p* < 0.001 for both comparisons) (Figure 5A). Similar results were observed using the weighted UniFrac distance algorithm (*p* = 0.032 and *p* = 0.042, respectively) (Figure 5B). However, no significant difference in beta diversity was observed between the hypertrophy and non-hypertrophy groups (*p* = 0.069 and *p* = 0.856 for unweighted and weighted UniFrac, respectively).

These findings showed that individuals with hypertension, regardless of the presence of LVH, share similar gut microbial communities, whereas significant differences exist when compared with healthy individuals.

### 3.4 Analysis of community structure differences between groups

**Figure 6:**
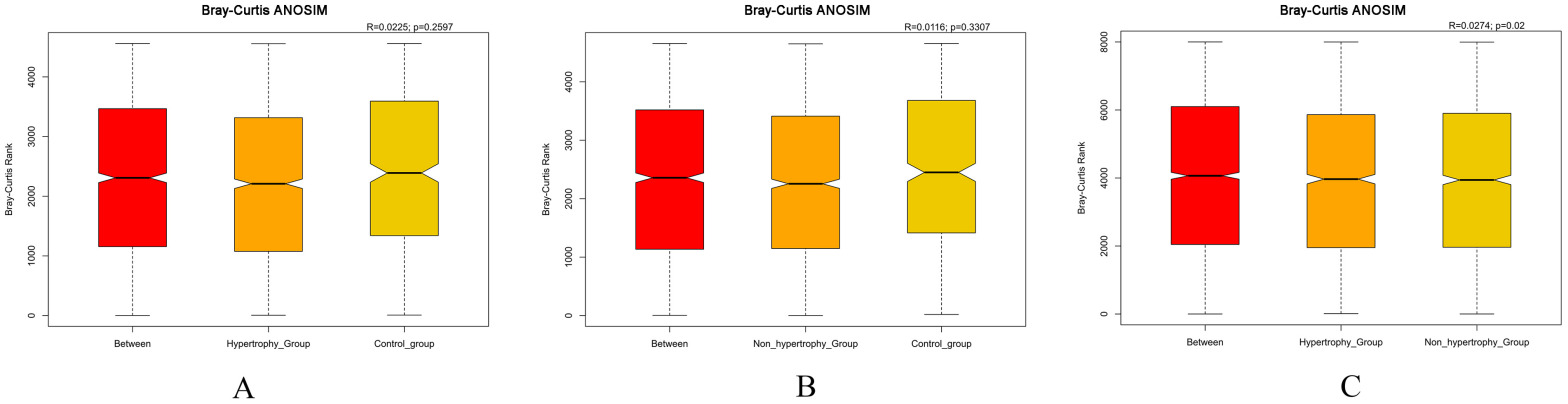
Bray–Curtis ANOSIM differential analysis among different groups (**A**: hypertrophy group vs. control group, *R* = 0.0225, *p* = 0.2597; **B**: non-hypertrophy group vs. control group, *R* = 0.0116, *p* = 0.3307; **C**: hypertrophy vs. non-hypertrophy group, *R* = 0.0274, *p* = 0.02).

A significance analysis of community structure differences between groups was conducted based on the ranked Bray–Curtis distance values. The results showed statistically significant differences in microbial community structure between the hypertrophy and non-hypertrophy groups (*p* = 0.019) (Figure 6C). However, no significant differences were observed between the hypertrophy and control groups or between the non-hypertrophy and control groups (*p* = 0.260 and *p* = 0.331, respectively) (Figures 6A,B).

### 3.5 Analysis of microbiota composition between the three groups

#### 3.5.1 Analysis of the relative abundance of microbiota between groups

**Figure 7:**
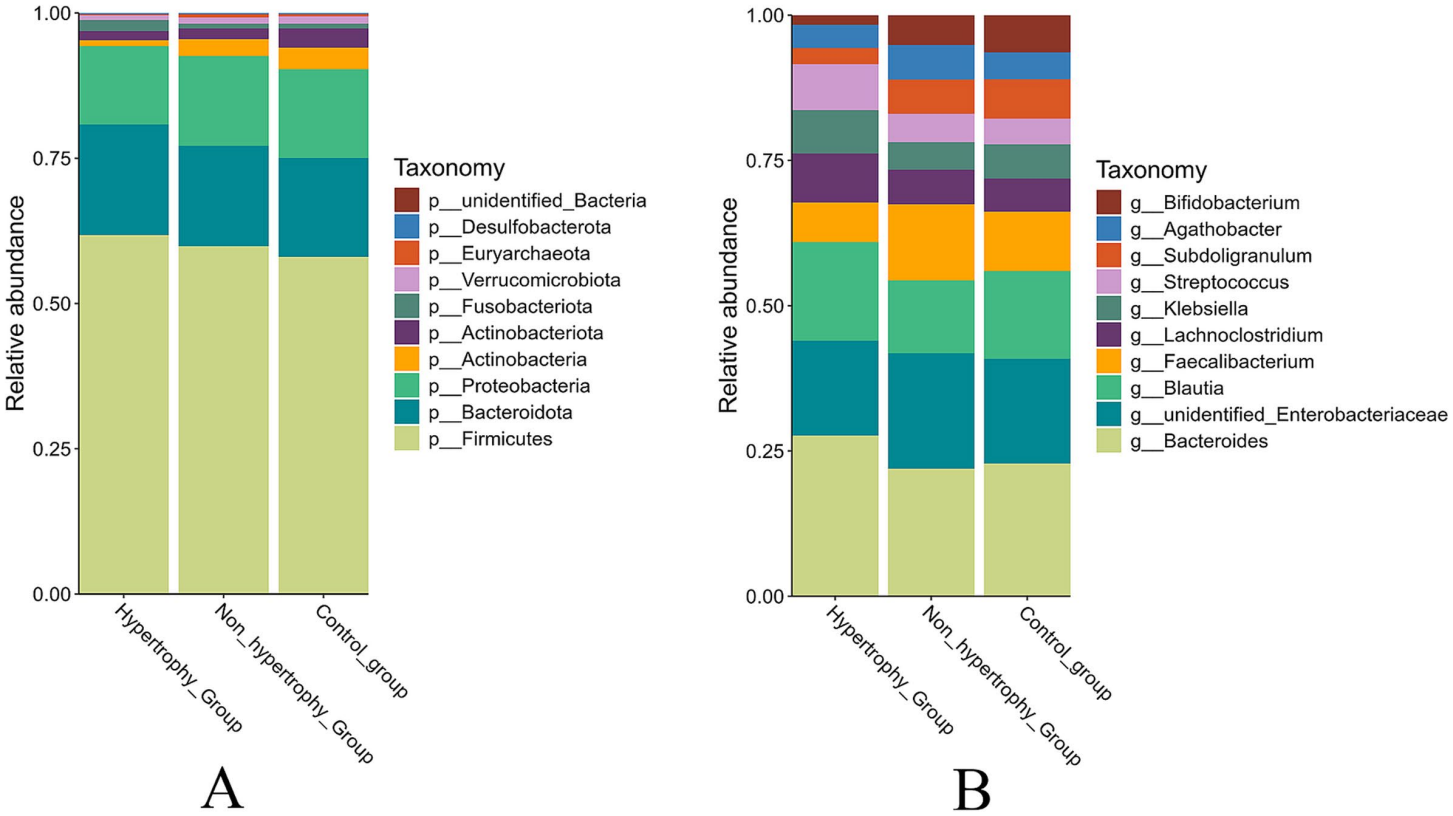
The relative abundance of taxonomy of the intestinal microbiota among different groups (**A**: phylum level; **B**: genus level).

The top 10 most abundant taxa at both the phylum and genus levels were selected to generate cumulative histograms, which visually illustrated the average relative abundance of dominant taxa across the sample groups. At the phylum level, Firmicutes, Bacteroidetes, and Proteobacteria were predominant within the gut microbiota. A significant reduction in the relative abundance of Actinobacteria was observed in the hypertrophy group compared to the control group (*p* = 0.049) (Figure 7A). At the genus level, the microbial composition in the non-hypertrophy group more closely resembled that of the control group (Figure 7B).

#### 3.5.2 Differential species analysis between groups

**Figure 8:**
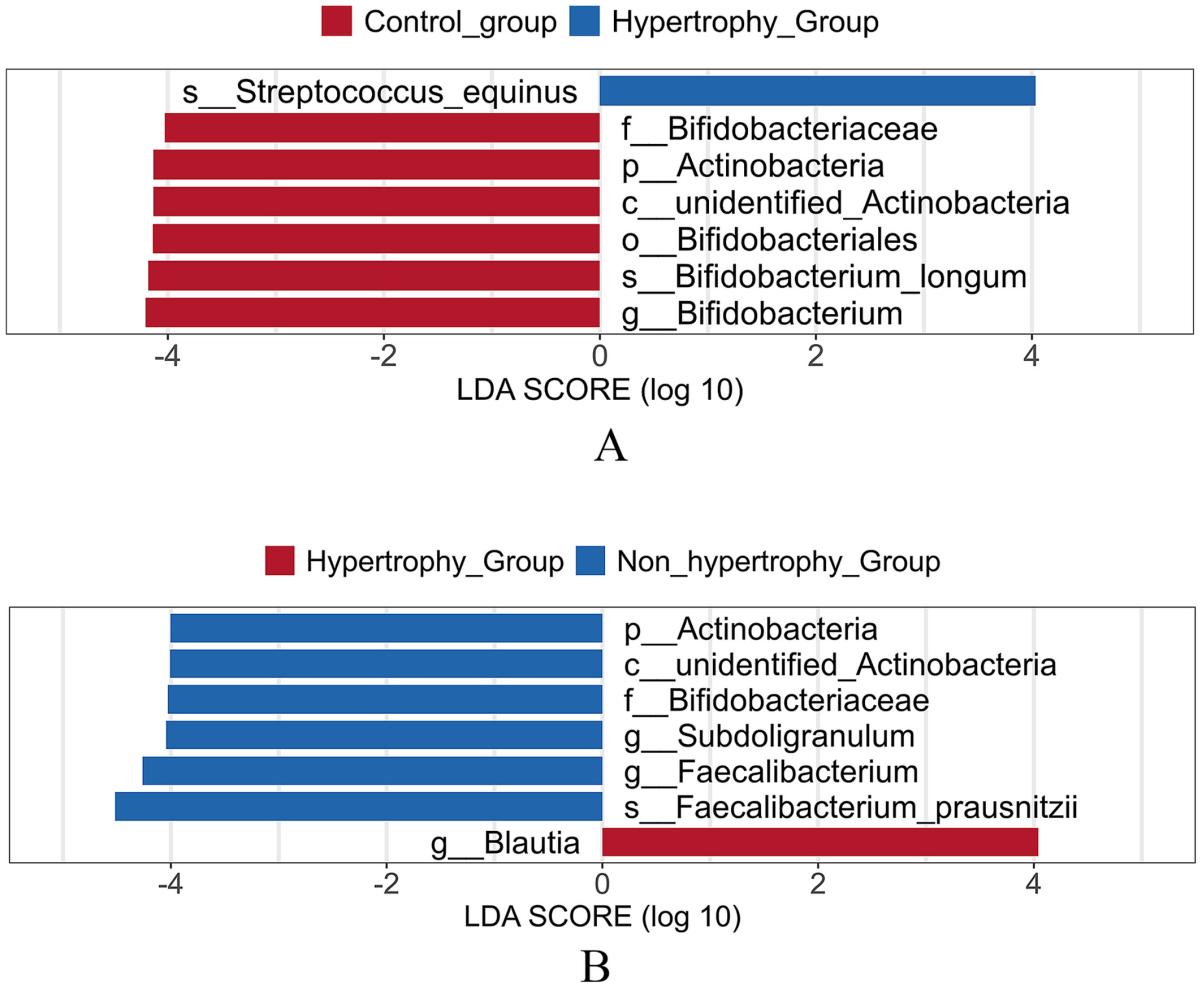
Linear discriminant analysis effect size (LEfSe) analysis of different species of intestinal flora among different groups (**A**: hypertrophy group vs. control group; **B**: hypertrophy group vs. non-hypertrophy group).

The LEfSe method was applied to identify differential bacterial taxa among the three groups. Higher abundances of *Bifidobacterium* and *Bifidobacterium longum* were observed in the control group at the genus and species levels, respectively. At the species level, an increased enrichment of *Streptococcus equinus* was found in the hypertrophy group (*p* < 0.05) (Figure 8A). When compared with the non-hypertrophy group, the hypertrophy group exhibited greater enrichment of *Blautia* at the genus level. Conversely, patients in the non-hypertrophy group exhibited significantly higher relative abundances of Actinobacteria (phylum level), Bifidobacteriaceae (family level), *Subdoligranulum* and *Faecalibacterium* (genus level), and *Faecalibacterium prausnitzii* (species level) (*p* < 0.05) (Figure 8B).

### 3.6 Random forest analysis

**Figure 9:**
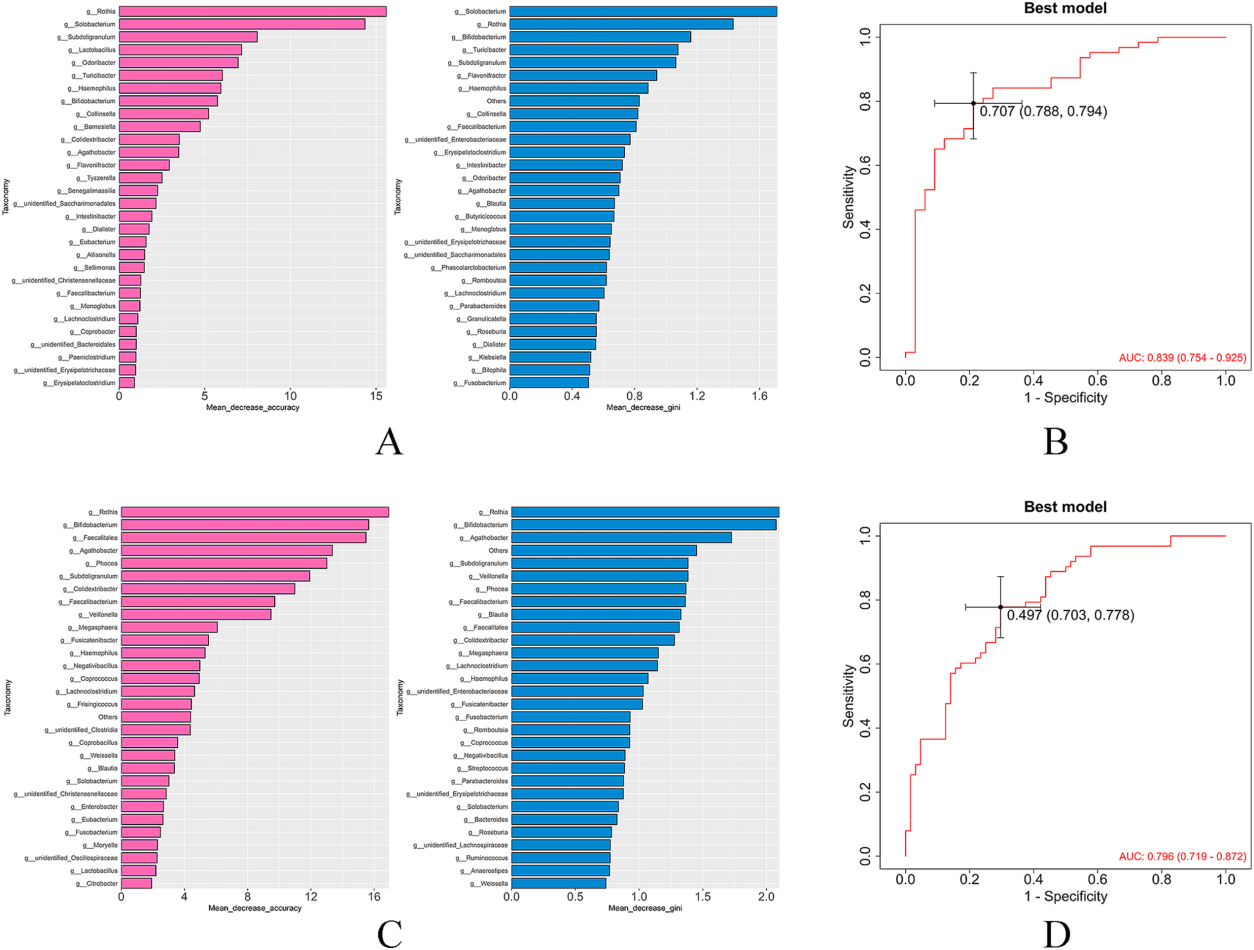
Ranking of the importance of bacteria and ROC curve for diagnosing LVH of based on random forest model (**A,B**: hypertrophy group vs. control group; **C,D**: hypertrophy group vs. non-hypertrophy group).

The mean decrease accuracy and mean decrease gini metrics were used to compare and rank the importance of gut bacterial species associated with hypertension and LVH (Figures 9A,C). A random forest prediction model was constructed based on the gut microbiota profiles.

The results demonstrated that the gut microbiota exhibited substantial diagnostic value in identifying hypertension with LVH. The model achieved an area under the curve (AUC) of 0.839 for distinguishing patients in the hypertrophy group from healthy individuals (Figure 9B), and an AUC of 0.796 for distinguishing patients in the hypertrophy group from those in the non-hypertrophy group (Figure 9D), indicating strong predictive performance.

## 4 Discussion

Hypertension is recognized as the most common cause of pathological myocardial hypertrophy and represents a significant independent risk factor for adverse cardiovascular events and mortality. In this study, uric acid levels were notably higher in patients with hypertension and LVH. Elevated serum uric acid has been associated with increased risk of hypertension and microalbuminuria, whereas the modulation of gut microbiota dysbiosis reduces inflammation, lowers serum uric acid levels, and improves renal function (Bjornstad et al., 2019; Liu et al., 2025). Hyperhomocysteinemia is frequently observed in individuals with hypertension. Impaired homocysteine metabolism and clearance, along with abnormal methylation and transsulfuration processes, contribute to cellular injury and organ dysfunction, which are key mechanisms underlying hypertension (Weber et al., 2016). Furthermore, abnormalities in bile acid and lipid metabolism represent another common pathological feature in hypertensive populations. A study conducted in Japan demonstrated that gut microbiota dysbiosis significantly affects both blood pressure regulation and lipid metabolism (Takagi et al., 2020). In this study, patients with hypertension and LVH showed significantly elevated triglyceride levels and reduced HDL-C levels.

Previous studies have reported that stress overload in individuals with hypertension induces mechanical stretching of cardiomyocytes, thereby activating intracellular signaling pathways that lead to the expression of genes and the synthesis of structural proteins such as actin and myosin (Han et al., 2024). However, the development of LVH in hypertensive individuals is not solely attributable to hemodynamic overload resulting from elevated blood pressure. Clinical observations have shown that in some cases, the hypertrophic myocardium remains unremitted despite long-term, well-controlled blood pressure (Devereux et al., 2004). Furthermore, animal studies have demonstrated that spontaneously hypertensive rats can develop LVH prior to the onset of elevated blood pressure (Kundu et al., 2015).

An increasing body of evidence suggests that gut microbiota contribute significantly to the pathogenesis and progression of hypertension (Avery et al., 2021). Prior studies have reported reduced alpha diversity of the gut microbiota in individuals with hypertension, as shown by a lower Shannon index, which has been found to be negatively correlated with blood pressure. However, no significant differences were observed in the Simpson, ACE, or Chao1 indices (Cai et al., 2023; Tsiavos et al., 2024). In contrast, the present study did not identify any significant differences in alpha diversity among the groups. This discrepancy may reflect the stage-specific microbial dynamics: early hypertension may exhibit diversity loss, whereas LVH development (as in our cohort) could be driven by specific pathobiont expansion rather than global diversity collapse. Additionally, examination of baseline characteristics showed a higher proportion of male participants in the hypertensive group with LVH, and it is possible that sex-based differences may influence the composition of the gut microbiota (Sharma et al., 2025).

Prior studies have shown that individuals with hypertension frequently exhibit gut microbiota dysbiosis, characterized by an overgrowth of potentially pathogenic taxa and a reduction in beneficial microbial populations. This dysbiosis may contribute to the pathogenesis of hypertension and the progression of target organ damage. For instance, this study demonstrated that the relative abundances of Firmicutes and Bacteroidetes were elevated in patients with hypertension and LVH, whereas Actinobacteria were significantly reduced.

Microbial imbalances may impair intestinal barrier function, facilitating the translocation of endotoxins—such as lipopolysaccharides (LPS)—into the systemic circulation. This process can trigger a systemic inflammatory response, thereby contributing to the development of multiple conditions, including insulin resistance, dyslipidemia, and cardiovascular disease (Moludi et al., 2020). LPS upregulates the expression of inflammation-related genes and increases circulating levels of inflammatory mediators, such as tumor necrosis factor-α and interleukin-6, which may inhibit mitochondrial fatty acid oxidation in cardiomyocytes and consequently impair myocardial function (Zou et al., 2022). Additionally, LPS may promote the progression of atherosclerosis by activating the Toll-like receptor 4 signaling pathway and enhancing platelet aggregation (Zhou et al., 2018). These inflammatory responses are considered potential mechanisms contributing to the development of LVH in individuals with hypertension.

Furthermore, the intestinal microbiota can produce a rich group of metabolites, whose levels are closely related to human health. Gut microbiota-derived metabolites, including short-chain fatty acids (SCFAs), bile acids, and trimethylamine N-oxide (TMAO), have also been strongly linked to cardiovascular disease. SCFAs, generated through the fermentation of dietary fiber by gut microbiota, possess anti-inflammatory and metabolic regulatory properties (Chen et al., 2020). These metabolites attenuate myocardial remodeling associated with hypertension by activating G protein-coupled receptors, such as GPR43 and GPR109A (Kaye et al., 2020). Consequently, reductions in SCFA levels due to microbial dysbiosis may represent an additional contributing factor in the development of LVH in hypertensive individuals. TMAO, another key microbial metabolite, has been implicated in vascular inflammation, foam cell formation, atherogenesis, and increased risk of platelet overactivation and thrombosis (Koeth et al., 2019; Zhu et al., 2016; Seldin et al., 2016). It is hypothesized that changes in the composition of the gut microbiota may exacerbate LVH by altering the levels of such metabolites. In an animal model study conducted by Li et al., dietary-induced elevation of TMAO levels was found to promote ventricular hypertrophy and fibrosis via the Smad3 signaling pathway. Inhibition or reduction of TMAO production through modulation of gut microbiota may thus represent a potential therapeutic strategy for the prevention and treatment of ventricular hypertrophy (Li et al., 2019).

Currently, it is proposed that the gut microbiota may serve as a promising therapeutic target for hypertension management in the future (Mahgoup, 2025). Modulation of the composition and function of the gut microbiota through dietary interventions or probiotic supplementation has been reported to exert beneficial effects on both hypertension and LVH (Xue et al., 2021; Mähler et al., 2020). Additionally, fecal microbiota transplantation (FMT), an emerging therapeutic strategy, has demonstrated the potential to restore microbial balance and reduce blood pressure in individuals with hypertension (Fan et al., 2022). In a hypertensive rat model treated with angiotensin receptor blockers, FMT significantly lowered systolic and mean arterial pressures following ARB administration, and further reduced vascular injury and collagen deposition (Li et al., 2024). These findings propose that modulation of the gut microbiota via FMT may confer protective effects against hypertensive LVH.

In summary, our findings reported that patients with hypertension and LVH exhibited a higher prevalence of metabolic disturbances, including hyperuricemia, hyperhomocysteinemia, and hypertriglyceridemia, along with a greater susceptibility to renal impairment and more pronounced cardiac remodeling. Significant changes in the abundance, diversity, and structural composition of the gut microbiota were observed in patients with hypertension and LVH. *Bifidobacterium* were more abundant among healthy individuals. In contrast, patients with hypertension and LVH demonstrated a marked reduction in the relative abundance of Actinobacteria and increased enrichment of *Streptococcus equinus*. The microbial structure in patients with hypertension without LVH more closely resembled that of healthy individuals. The above findings revealed that gut flora intervention may be an important target for the prevention and treatment of hypertension-related cardiac target organ damage in the future. Given the current encouraging results, more cohorts should be collected in the future to continue verifying the differential species or conduct metagenomic analysis to achieve precise intervention of the gut microbiota in hypertensive individuals with LVH.

Several limitations should be acknowledged in this study. First, considerable sex differences were present among the groups at baseline, and stratification by age and comorbidities was not performed, which may have introduced potential confounding effects. Second, although all participants were recruited from the same local community and thus shared broadly similar dietary patterns, we did not perform quantitative dietary assessments; therefore, residual dietary variability could have influenced the results. Third, the absence of fecal and plasma metabolomics analysis limited further investigation into the mechanistic associations between gut microbiota dysbiosis and the development of LVH in individuals with hypertension.

## 5 Conclusion

BMI, uric acid, triglycerides, homocysteine, alkaline phosphatase, and γ-glutamyl transferase levels were significantly elevated in patients with hypertension and LVH, and the composition of the gut microbiota differed significantly from that observed in hypertensive patients without LVH and in healthy individuals. These findings provide new insights into the potential relationship between hypertension-related LVH and gut microbiota, and may inform future strategies for the prevention and treatment of cardiac target organ damage associated with hypertension.

## Data Availability

The raw sequence data reported in this paper have been deposited in the Genome Sequence Archive (Genomics, Proteomics & Bioinformatics 2025) in National Genomics Data Center (Nucleic Acids Res 2025), China National Center for Bioinformation/Beijing Institute of Genomics, Chinese Academy of Sciences (GSA-Human: HRA014282) that are publicly accessible at https://ngdc.cncb.ac.cn/gsa-human, (Zhang et al., 2025; CNCB-NGDC Members and Partners, 2025).

## Footnotes

^1^ https://github.com/OpenGene/fastp

^2^ http://ccb.jhu.edu/software/FLASH/

^3^ https://github.com/torognes/vsearch/

^4^ http://www.drive5.com/uparse/

^5^ http://www.arb-silva.de/

^6^ https://cloud.metware.cn
